# Characterization of the complete mitochondrial genome of *Spirometra decipiens* (Cestoda: Diphyllobothriidae) from China

**DOI:** 10.1080/23802359.2019.1659117

**Published:** 2019-09-06

**Authors:** Yue Xie, Yingxin Li, Xiaobin Gu, Senzhao Zhang, Yunjian Liu, Lu Wang, Youle Zheng, Xuan Zhou, Zhicai Zuo, Guangyou Yang

**Affiliations:** a Department of Parasitology, College of Veterinary Medicine, Sichuan Agricultural University, Chengdu, China;; b Institute of Animal Genetics and Breeding, College of Animal Science and Technology, Sichuan Agricultural University, Chengdu, China;; c Key Laboratory of Animal Disease and Human Health of Sichuan Province, College of Veterinary Medicine, Sichuan Agricultural University, Chengdu, China

**Keywords:** *Spirometra decipiens*, sparganosis, mitochondrial genome, phylogenetic analysis

## Abstract

The plerocercoid larvae (spargana) of *Spirometra decipiens* (Cestoda: Diphyllobothriidae) can parasitize humans, causing the zoonotic sparganosis. In this study, the complete mitochondrial genome of this tapeworm was determined using an Illumina sequencing platform. The entire genome was 13,642 bp in length and contained 12 protein-coding genes, 22 transfer RNAs, two ribosomal RNAs, and two non-coding regions. The phylogeny indicated that *S. decipiens* was closely related to *Spirometra erinaceieuropaei* and supported the monophyletic relationships between *Spirometra*, *Diphyllobothrium*, and *Diplogonoporus* within the Dipyllobothriidae. These results should contribute to a better understanding of the phylogenetic position of this species.

The plerocercoid larvae (spargana) of *Spirometra* spp. (Cestoda: Diphyllobothriidae) can parasitize humans, causing the zoonotic sparganosis (Zhang et al. 2017). Although human sparganosis is mostly attributed to *Spirometra erinaceieuropaei* and *Spirometra mansonoides*, recent reports show that *Spirometra decipiens* is also responsible for sparganosis in Asia and has caused 15 clinic cases in Korea (Cui et al. 2011; Jeon et al. 2015). Consequently, *S. decipiens* was added to the causative agent list of human sparganosis (Jeon et al. 2016). As with other *Spirometra* spp., current diagnosis of *S. decipiens* infection is typically based on morphological identification of spargana. However, it usually becomes difficult when identification of the larvae is performed among some possible cross-infected larvae of congeneric species including *S. erinaceieuropaei* and *Spirometra mansoni* and related species of *Diphyllobothrium*. Therefore, there is an urgent need for obtaining a more efficient and reliable approach to identify *S. decipiens*. Mitochondrial DNA (mtDNA) is proven to be valuable complementary tools and has been widely used for species-specific identification of many zoonotic parasites (Le et al. 2002; Hu and Gasser 2006). Here, we reported the complete mitochondrial genome sequence of *S. decipiens* from China.

The parasite samples were obtained from an infected farmed dog at a slaughterhouse at Wenjiang, Sichuan Province of China, after treatment with praziquantel. After morphological identification, the tapeworm specimens (*n* = 2) were identified as *S. decipiens* according to the taxonomic key of Faust (1929) and molecular confirmation by amplification and sequencing of the mitochondrial *cox1* and *nad3* (Jeon et al. 2016). One tapeworm was used for DNA extraction, and another was archived in the Parasitological Museum of Sichuan Agricultural University (Sichuan, China) under collection numbers XY2018_4. Total mtDNA was isolated and sequenced using the Illumina HiSeq platform (Novogene, Tianjin, China). The mitogenome assembly was carried out with MITObim (Hahn et al. 2013), and gene annotation was performed by MITOS (Bernt et al. 2013).

The complete mtDNA of *S. decipiens* was 13,642 bp in length (GenBank accession no. MN121695) and encoded 12 protein-coding genes, 22 tRNAs, and two rRNAs. All genes were unidirectionally transcribed on the same strand. Among the 12 protein-coding genes, except *cox1* and *nad3* deduced to use an incomplete stop codon ‘T’, the rest were predicted to use the typical TAA or TAG as the stop codons. Twenty-two tRNA genes ranged from 57 bp (tRNA-Arg) to 70 bp (tRNA-Thr) in length. Both rRNAs were 730 bp (*12S*) and 973 bp (*16S*) in length, respectively, and located between tRNA-Thr and *cox2* with a separation by tRNA-Cys. Two large non-coding regions, namely NC1 (203 bp) and NC2 (174 bp), were placed between tRNA-Tyr and tRNA^(CUN)^-Leu and between *nad5* and tRNA-Gly, respectively.

A maximum-likelihood (ML) phylogeny was reconstructed on a concatenated amino acid dataset of 12 protein-coding genes from 48 flatworms, using one trematode *Schistosoma japonicum* as outgroup. This phylogeneric tree clearly showed that *S. decipiens* was closely related to *S. erinaceieuropaei*, regardless of isolate origins, with high bootstrap confidence and supported the monophyletic relationships between *Spirometra*, *Diphyllobothrium*, and *Diplogonoporus* within the Dipyllobothriidae (Figure 1).

**Figure 1.**
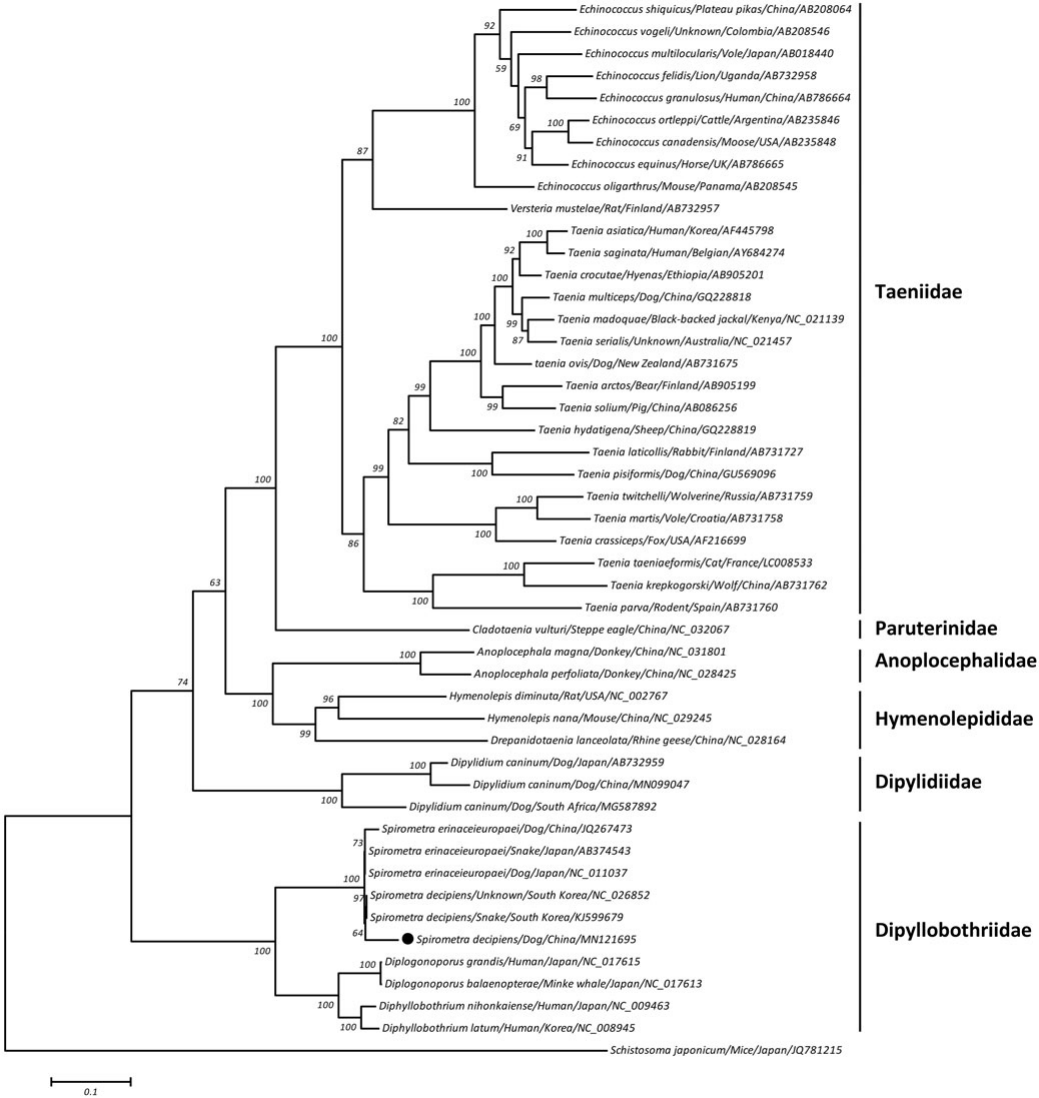
Maximum-likelihood (ML) tree inferred from concatenated amino-acid sequences of 12 mitochondrial protein-coding genes of *S. decipiens* and other related flatworms, utilizing MtArt model with 1000 bootstrap replications (<50% support not shown). The solid black circle represents the species in this study.
